# Remote Sensing Applications in Medicinal Plant Monitoring and Quality Assessment: A Review

**DOI:** 10.3390/s26082465

**Published:** 2026-04-16

**Authors:** Ziying Wang, Jinping Ji, Guanqiao Chen, Yuxin Fan, Jinnian Wang, Yingpin Yang, Xumei Wang

**Affiliations:** 1 School of Pharmacy, Xi’an Jiaotong University, Xi’an 710061, China; ziy_wang@stu.xjtu.edu.cn (Z.W.); jijinping1003@stu.xjtu.edu.cn (J.J.);; 2 Tandon School of Engineering, New York University, New York, NY 11201, USA; 3 School of Geography and Remote Sensing/Institute of Aerospace Remote Sensing Innovations, Guangzhou University, Guangzhou 510006, China; jnwang@gzhu.edu.cn; 4 Xi’an Botanical Garden of Shaanxi Province, Xi’an 710061, China

**Keywords:** medicinal plants, remote sensing, resources, monitoring, quality assessment, environmental stress

## Abstract

As a core resource of traditional Chinese medicine (TCM), medicinal plants are conventionally monitored and assessed using high-cost, low-efficiency methods. Remote sensing offers an efficient technical alternative for large-scale and dynamic evaluation. This study systematically reviewed the literature from 2005 to 2025, summarized remote sensing platforms, sensors, and data analytical methods, and specifically analyzed their applications in medicinal plant resource investigation, planting monitoring, stress monitoring, and TCM quality assessment. These studies mainly focus on resource surveys and quality analysis, targeting root and rhizome herbs. Integrated satellite-, UAV-, and ground-based remote sensing enables distribution mapping, growth retrieval, stress monitoring, and non-destructive quality evaluation in medicinal plants, achieving overall accuracies ranging from 80% to 100%. Currently, remote sensing applications in medicinal plants are evolving toward space–air–ground integration, multi-source data fusion, artificial intelligence empowerment, and multi-omics integration. However, they are constrained by complex wild habitats, difficulties in monitoring root herbs, spectral confusion, and limited model generalization. Future efforts should focus on establishing an integrated monitoring network, developing full-chain quality inversion models for geo-authentic herbs, building climate-adaptive cultivation systems, creating early pest–disease warning technologies, and deepening the integration of remote sensing and multi-omics to support the sustainable utilization and high-quality development of medicinal plant resources.

## 1. Introduction

Medicinal plants are among the most ancient and indispensable natural resources, with over 50,000 species used worldwide for medicinal purposes [1], providing primary healthcare support for 70–80% of the global population [2]. The global herbal medicine market, valued at USD 70.57 billion in 2023, is projected to exceed USD 328 billion by 2030 [3], highlighting both the medicinal importance and economic value of these resources. In addition, medicinal plants serve as key raw materials for modern pharmaceuticals, with more than half of approved drugs derived from plant-based compounds [4].

Despite their significance, medicinal plant resources face the dual challenges of supply and management. Wild populations are rapidly declining due to overexploitation, habitat loss, and climate change [5], posing significant threats to many rare species’ survival [6]. Although cultivation has become the primary strategy to alleviate pressure on wild resources, it is often conducted without standardized agronomic practices. Inconsistent management, poor traceability, and inadequate quality control significantly affect both yield and medicinal efficacy [7]. These challenges threaten the sustainable development of TCM and broader herbal medicine industries, underscoring the need for advanced and scalable monitoring technologies.

Remote sensing has emerged as a transformative technology for addressing these challenges. With its broad spatial coverage, rich spectral information, temporal continuity, and capacity for rapid, non-destructive data acquisition [8], it enables large-scale, objective, and repeatable observations beyond conventional field surveys. These advantages have been widely applied in medicinal plant research, including species distribution mapping, planting area estimation, and growth monitoring [9]. More recently, unmanned aerial vehicle (UAV)-based remote sensing, combined with deep learning, has shown strong potential for medicinal plant monitoring, with advanced models such as Rose-Mamba-YOLO [10] and Succulent-YOLO [11] improving the accuracy of plant detection and classification.

Nevertheless, existing reviews have primarily focused on species identification and classification [8,9,12], with limited attention to growth monitoring, environmental stress, and quality assessment. This imbalance constrains the use of remote sensing as a holistic tool for intelligent medicinal plant cultivation management. To address this gap, this review synthesizes recent advances in remote sensing applications across four core domains: (1) medicinal plant resource investigation, (2) planting monitoring, (3) stress monitoring, and (4) quality assessment of TCM. Furthermore, considering the diversity of planting patterns and habitat characteristics, this review provides guidance on selecting appropriate remote sensing platforms, sensor types, and analytical methods. Finally, key technical challenges are identified, and future research priorities and implementation strategies are outlined.

## 2. Remote Sensing Technology and Medicinal Plants

### 2.1. Research and Development Trends

Remote sensing technology was applied to medicinal plant research as early as the beginning of this century, when satellite imagery was used to identify and estimate the reserves of *Panax notoginseng* and *Panax ginseng* [13,14]. In 2007, Xie et al. pioneered the use of UAV remote sensing for medicinal plant resource surveys and subsequently conducted field investigations of the wild endangered species *Ferula sinkiangensis* [15,16]. These early efforts provided critical technical support for the fourth National Chinese Medicinal Resources Census (2011–2022), demonstrating the practical feasibility of remote sensing for large-scale medicinal plant investigation and monitoring [17]. Recent advances in spatiotemporal resolution and analytical accuracy have accelerated the application of remote sensing in medicinal plant research, covering over 100 species [18,19,20,21].

To systematically characterize research trends, a comprehensive literature review was conducted following a structured two-step keyword search strategy in the Web of Science and China National Knowledge Infrastructure (CNKI) databases. In the first step, remote sensing-related terms were used (e.g., “remote sensing”, “unmanned aerial vehicle”, “hyperspectral imaging”, “multispectral remote sensing”, and “time-series remote sensing”). In the second step, medicinal plant-related terms were applied (e.g., “medicinal plant”, “herbal medicine”, “aromatic plant”, and “traditional Chinese medicine”). The search covered the period from January 2000 to December 2025, with no restrictions on document type or language.

All retrieved records were screened based on predefined inclusion criteria (Table 1). A total of 365 records were initially identified, and full texts were subsequently obtained from major scientific databases. After removing irrelevant studies through systematic screening, 296 publications were retained for further analysis.

**Figure 1:**
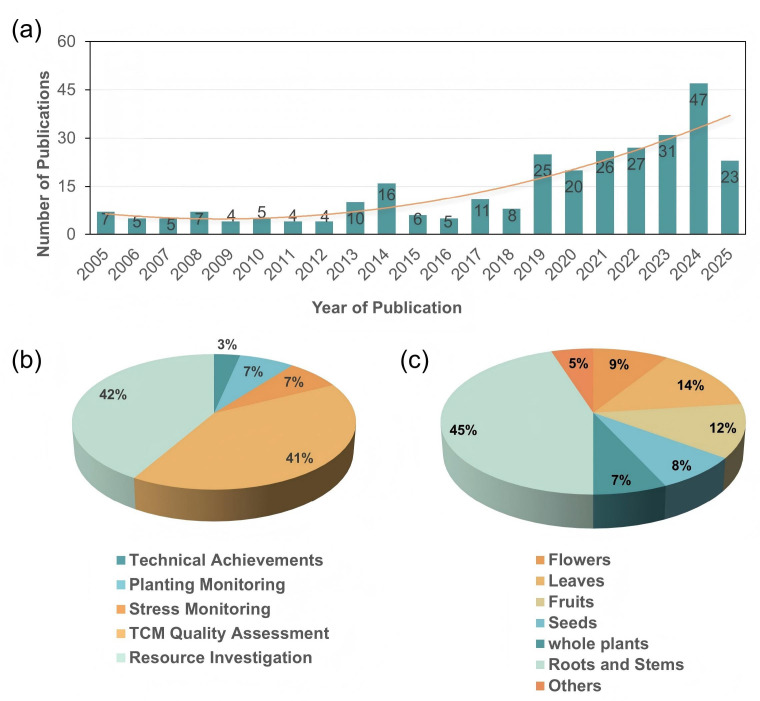
Literature statistics of remote sensing research on medicinal plants over the past two decades. (**a**) Annual number of research papers published on the review topic from 2005 to 2025; (**b**) proportion of each application scenario across all publications; (**c**) statistics of types of medicinal plants in existing studies (categorized by medicinal parts). Notes: the category “Flowers” includes flowers and inflorescences; “Leaves” includes blades and scale leaves; “whole plant” includes whole plants and aerial parts; “Roots and Stems” includes roots, rhizomes, root tubers, rootstocks, tubers, bulbs, stems, and cauline leaves; “Fruits” includes fruits and pericarps; “Others” includes resins and related materials.

The bibliometric analysis (Figure 1a) indicates a rapid increase in remote sensing research on medicinal plants over the past two decades. In terms of application domains (Figure 1b), resource investigation and TCM quality assessment were the most studied topics, accounting for 42% and 41% of publications, respectively. These were followed by planting monitoring (7%), stress monitoring (7%), and technological applications (3%). Regarding classification by medicinal parts (Figure 1c), most studies focused on root- and rhizome-based medicinal plants (45%), highlighting their primary research importance. Other categories included leaves (14%), fruits (12%), flowers (9%), seeds (8%), whole plants (7%), and others (5%). A detailed inventory of the reviewed studies, including plant species, families, medicinal parts, remote sensing platforms, study regions, and application scenarios, is provided in the Supplementary Files.

### 2.2. Platforms and Sensors

The selection of remote sensing platforms for medicinal plant monitoring is primarily determined by spatial scale and observation objectives. Satellite-based remote sensing, particularly medium- (10–30 m) [22,23] and high-resolution (meter to sub-meter) [24,25] data, is well-suited for large-scale applications such as resource surveys, distribution mapping, and growth monitoring, with its ability to provide large-area, periodic, and cost-effective data acquisition. In contrast, UAVs provide flexible, high-resolution observations (decimeter to centimeter level) over targeted areas, enabling detailed monitoring at the field and individual plant scales [26]. Ground-based and laboratory approaches, therefore, serve as essential complements, allowing direct measurements of specific plant organs (e.g., roots, stems, and seeds) and supporting applications such as component analysis and authenticity identification.

In terms of sensor selection, multispectral and hyperspectral sensors are the most widely used. Multispectral sensors, characterized by low cost and broad availability, are commonly applied in satellite- and UAV-based monitoring for large-scale tasks. Hyperspectral sensors, by contrast, provide higher spectral resolution and are more effective for retrieving physicochemical properties and enabling detailed characterization of plant status [27,28].

Despite the advances in remote sensing, monitoring medicinal plants faces distinct challenges due to their unique growth characteristics. Many medicinal plants, particularly root- and rhizome-based species, have subterranean organs that cannot be directly assessed through canopy-level observations. Additionally, the fragmented distribution of wild medicinal plants and the complex environments they inhabit, such as shaded forest understories, further complicate remote sensing efforts. These factors demand more targeted strategies that address the specific monitoring needs of different plant types. For root- and rhizome-type species, integrating canopy spectral information with ground-based measurements and biochemical data offers a feasible pathway for indirectly assessing belowground conditions. For forest-based medicinal plants under shaded environments, incorporating structural information is essential to mitigate canopy occlusion effects. In this context, light detection and ranging (LiDAR) provides unique advantages in capturing subcanopy structural characteristics and shows strong potential for detecting understory features and supporting indirect inference of belowground conditions [29]. Despite its widespread use in forestry and geological applications, it remains underutilized in plant research, representing a promising direction for future exploration [30].

### 2.3. Data Analysis Methods

The choice of remote sensing data analysis methods is determined by the target application. For resource monitoring, pixel-based classification and object-based image analysis (OBIA) are used to extract medicinal plants and estimate coverage [9]. Pixel-based methods are efficient but lack spatial context and are prone to noise. OBIA improves accuracy by grouping contiguous pixels but requires more computational power. Pixel-based methods work well for large-scale canopy coverage, while OBIA is better for fine-scale plant delineation. For growth monitoring, vegetation index inversion is used to assess plant health and growth dynamics [31,32]. Extracting key phenological parameters from the seasonal trajectories of vegetation indices also serves as an important approach for tracking growth stages [33].

For stress monitoring, hyperspectral data are analyzed to extract red-edge position and water indices for assessing plant water status, while thermal infrared data are used to retrieve canopy temperature and detect stress signals such as drought. For quality monitoring, hyperspectral data are analyzed through modeling techniques or spectral matching methods to evaluate the quality of medicinal materials [34].

Across these application domains, selecting an appropriate modeling method depends on factors such as data availability, task complexity, and the need for interpretability. Traditional machine learning methods (e.g., Partial Least Squares Regression [PLSR], Support Vector Machine [SVM], and decision tree-based algorithms) are particularly well-suited for tasks like single plant inversion and component inversion when training data are limited [35]. These methods perform reliably with small sample sizes, offer strong interpretability, and have modest computational demands. They are ideal when model transparency is crucial, but their main drawback lies in the reliance on manual feature engineering and their limited ability to capture complex, nonlinear spectral or spatial relationships [36].

In contrast, deep learning architectures, which learn hierarchical representations directly from raw data, are particularly effective in tasks like species recognition and multi-component retrieval, where nonlinear interactions among target analytes present challenges for traditional methods [37]. For instance, convolutional neural networks (CNNs) have demonstrated outstanding performance in species recognition, while attention-based models excel in component inversion tasks, where simultaneous retrieval of multiple analytes is required. However, deep learning methods are limited by their high demand for large annotated datasets and significant computational resources [38].

## 3. Application of Remote Sensing Technology in Medicinal Plants

**Figure 2:**
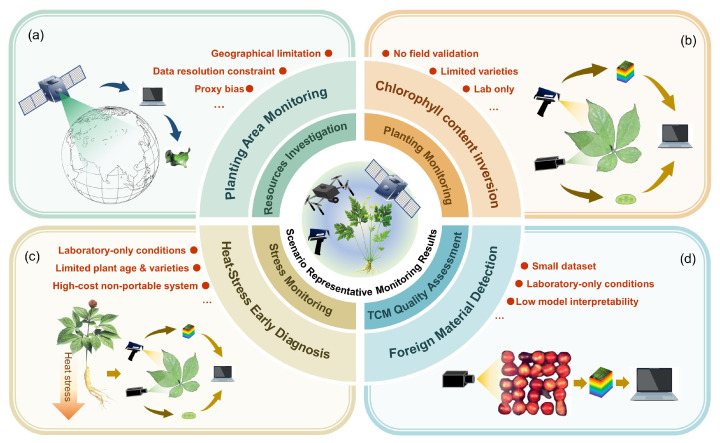
Representative monitoring results and limitations of medicinal plants across remote sensing application scenarios. (**a**) Remote sensing monitoring of spatiotemporal variations in *Panax notoginseng* planting area in Kaiyuan city using satellite data [39]. (**b**) Rapid and non-destructive retrieval of chlorophyll content in ginseng leaves based on spectral reflectance data [40]. (**c**) Early diagnosis of heat-stressed ginseng plants using fluorescence hyperspectral imaging [41]. (**d**) Precise identification of exogenous impurities in semen ziziphi spinosae using hyperspectral imaging [42].

Based on relevant research both domestically and internationally, this review examines the application of remote sensing in medicinal plant monitoring in four key areas: resource investigation, planting monitoring, stress monitoring, and quality assessment in TCM (Figure 2 presents representative monitoring results for the four core remote sensing application scenarios for medicinal plants and illustrates their major limitations with examples). In the resource investigation section, medicinal plant resources are categorized into wild and cultivated types according to their distinct distribution characteristics, outlining their respective distribution patterns and corresponding remote sensing methodologies. Planting monitoring primarily addresses plant physiological status and environmental conditions, summarizing current research advances and techniques. Regarding stress monitoring, the review covers applications related to temperature, water, heavy metals, pests, and diseases, and provides a summary of methods and recommendations. Finally, in the section on quality assessment of TCM, the mechanisms and existing methodologies for both species identification and component inversion are reviewed.

### 3.1. Resource Investigation

**Figure 3:**
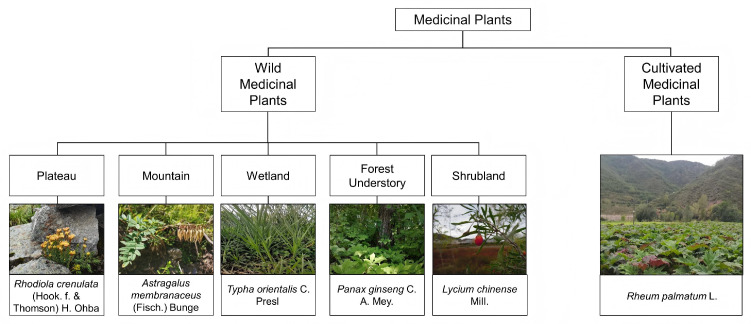
Classification of medicinal plant resources and associated habitat types, with representative species for wild and cultivated systems.

Medicinal plant resources can be broadly classified into wild and cultivated types based on their origin and management (Figure 3). Wild medicinal plants occur in diverse natural habitats, including plateaus, mountains, shrubland, forest understories, and wetlands, and are typically associated with specific ecological niches. In contrast, cultivated medicinal plants are managed under organized planting systems. These differences in resource type and habitat characteristics provide the basis for subsequent remote sensing strategies. These fundamental differences not only determine resource distribution characteristics but also critically influence the selection, performance, and limitations of remote sensing approaches, forming the basis for differentiated monitoring strategies.

#### 3.1.1. Wild Medicinal Plant Resources

When selecting remote sensing data for surveying wild medicinal plant resources, their ecological heterogeneity, sparse distribution, and complex background interference must be explicitly considered. These characteristics pose significant challenges to species discrimination and mapping accuracy. Previous studies demonstrate that medium- to low-resolution imagery can support large-scale surveys. Chen et al. [14] used enhanced thematic mapper + (ETM+) imagery combined with field sampling verification to map Jilin ginseng, achieving over 90% accuracy. However, such approaches are generally limited to estimating potential or habitat-level distributions, as mixed pixel effects hinder reliable species-level discrimination.

In contrast, Bahdu [43] investigated the planting area of *Carthamus tinctorius* L. using multi-resolution data sources, including WorldView-2, ZY-3, and ZY-1 02C imagery. The results showed that high-resolution data significantly improved overall classification accuracy compared to medium-resolution imagery. However, reliable automatic identification of the species remained challenging, likely due to spectral similarity with surrounding vegetation and the influence of fragmented planting patterns.

At finer scales, UAV-based approaches offer substantial advantages. Ding [44] combined centimeter-level drone imagery with a Mask Region-Based Convolutional Neural Network (Mask R-CNN) model to identify *Lamiophlomis rotata*, achieving high detection performance (AP > 80%). The use of UAVs equipped with high-resolution sensors allows for the acquisition of finely detailed imagery over relatively large areas, significantly improving the accuracy of species identification at small vegetation scales. This enabled detailed analyses such as plant counts and yield estimation. Nevertheless, such methods are limited by spatial coverage, operational cost, and scalability, making them less suitable for regional applications.

Effective monitoring of medicinal plant resources requires multi-source data integration and hierarchical strategies, combining large-scale satellite observations with localized high-resolution validation. However, significant challenges remain. Limited spectral separability, sparse distribution, and high spatial heterogeneity constrain reliable species-level identification. At the same time, differences in acquisition time, spatial resolution, and sensor characteristics introduce geometric and spectral inconsistencies during data fusion, increasing processing complexity and reducing model robustness [45,46]. Therefore, addressing these limitations requires more integrated and ecologically informed approaches to improve both feature separability and model reliability.

#### 3.1.2. Cultivated Medicinal Plant Resources

Cultivated medicinal plants are generally easier to study than their wild counterparts, as they are centrally distributed and systematically documented. Medium-resolution satellite imagery has proven effective for large-scale mapping of cultivated medicinal plants. For instance, Chen et al. [24] compared six supervised classification methods for mapping *Polygonatum cyrtonema* cultivation areas. They found that the SVM algorithm achieved the highest accuracy, while decision trees and random forests (RFs) have also been widely applied due to their robustness and efficiency. These results indicate that reliable large-scale mapping can be achieved using moderate-resolution data combined with relatively simple machine learning approaches.

The integration of multi-source and multi-temporal data helps enhance the efficiency and accuracy of large-scale crop identification. He et al. [47] improved mapping accuracy for *Artemisia argyi* by integrating multi-temporal GF-1 and GF-6 data with red-edge and violet band information. Zhuo et al. [48] demonstrated that, for *Glycyrrhiza uralensis*, custom vegetation indices (e.g., the Normalized Perennial Vegetation Index [NDPVI] and Normalized Wilting Index [NDVWI]) can reduce spectral confusion with other crops.

Therefore, remote sensing monitoring of cultivated medicinal plants should be centered on medium-satellite data combined with phenological information, custom vegetation indices, and multi-temporal data integration. This strategy enables efficient and cost-effective large-scale mapping while maintaining satisfactory accuracy.

However, given the diverse cultivation patterns and ecological environments of different medicinal plants, it is necessary to develop tailored classification models for accurate identification and extraction of planting areas from remote sensing imagery. The data sources and methods from related studies are summarized in the table below (Table 2).

### 3.2. Planting Monitoring

#### 3.2.1. Growth Status

Accurate characterization of growth status is fundamental to ensuring the yield and quality of medicinal plants. Remote sensing technologies, including multispectral, hyperspectral, and radar sensors, provide multi-source data for assessing plant growth conditions. Multispectral data are widely used to estimate structural parameters such as leaf area index and vegetation cover, whereas hyperspectral data, owing to their high spectral resolution, enable the retrieval of key physiological and biochemical traits, including chlorophyll and nitrogen content. Radar data (e.g., Synthetic Aperture Radar [SAR]), in contrast, are sensitive to canopy structure and moisture conditions and offer all-weather observation capabilities, thereby complementing optical sensors. These variables are typically quantified through inversion models that link remote sensing signals to plant attributes [51].

Chlorophyll content serves as a key indicator for assessing photosynthetic capacity, growth status, and nutrient condition. The spectral vegetation index method is commonly employed for this purpose, using indices such as the normalized difference vegetation index (NDVI) and the ratio vegetation index (RVI) to quantify the relationship between the spectral characteristics of medicinal plants and their chlorophyll content from different analytical perspectives. Numerous effective vegetation indices have been developed to date, each with its own strengths and limitations. Researchers must therefore tailor their selection to the specific traits of the medicinal plants under study when choosing an appropriate vegetation index.

Recent studies highlight the increasing integration of hyperspectral data with advanced modeling techniques. For instance, Wang et al. [40] developed multiple prediction models for estimating chlorophyll content in *Panax ginseng* leaves, with the VI-SPA-RFR model achieving superior performance (RMSE = 1.1568; MAE = 0.9936). Similarly, Xu et al. [52] integrated UAV- and ground-based hyperspectral data with highly correlated vegetation indices to monitor chlorophyll dynamics in *Glycyrrhiza uralensis*, achieving high predictive accuracy (R^2^ = 0.95). Furthermore, machine learning methods such as RF, Extreme Gradient Boosting (XGBoost), and one-dimensional CNNs have demonstrated clear advantages in capturing nonlinear relationships in hyperspectral data, resulting in improved estimation accuracy [53,54]. These approaches also show potential for retrieving nutrient-related variables [55].

Despite these advances, research on the growth monitoring of medicinal plants remains limited. This is mainly constrained by the small and heterogeneous cultivation patterns, often under canopy conditions, which hinder reliable signal acquisition and reduce model transferability. In addition, current studies largely focus on general physiological indicators, while the retrieval of pharmacologically relevant compounds, particularly secondary metabolites, remains a key challenge.

#### 3.2.2. Growing Environment

Plant phenotypes emerge from the interaction between genetic background and environmental conditions, underscoring the importance of environmental monitoring in medicinal plant cultivation. Remote sensing provides an effective means to characterize key environmental variables that regulate plant growth. For example, soil-available silicon (SAS) and soil moisture (SM) contents are critical determinants for ginseng growth. Xu et al. [56] used hyperspectral imaging (HSI) technology combined with machine learning algorithms to build a non-destructive inversion model for SAS and SM, providing a practical basis for the non-destructive testing of Panax ginseng soil from the three provinces of northeast China. In addition, long-term time-series remote sensing technology can monitor the phenological changes of medicinal plants and their responses to climate factors.

Currently, remote sensing studies on the growth environment of medicinal plants remain limited. Systematic monitoring of key environmental drivers would not only support standardized cultivation and precision management of water and nutrients, but also enhance early warning of abiotic and biotic stresses. Strengthening research in this area is therefore essential to advance the application of remote sensing in medicinal plant cultivation. Representative studies are summarized in Table 3.

### 3.3. Stress Monitoring

#### 3.3.1. Temperature Stress

Temperature is an important environmental factor affecting plant growth and yield. Low-temperature stress can inhibit chlorophyll synthesis and reduce metabolic rates [57,58], whereas prolonged exposure to high temperatures accelerates leaf senescence, damages photosynthesis, and exacerbates oxidative stress [59]. Remote sensing technology enables the detection of temperature stress by capturing spectral variations under different thermal conditions. Several studies have established models to identify temperature-induced stress. Fu et al. [60] used UAV remote sensing to obtain vegetation indices under high-temperature and drought stress, constructed a drought resistance identification model using an SVM with an accuracy of 0.74, and effectively assessed the drought tolerance of *Boehmeria nivea*. Faqeerzada et al. [41] employed fluorescence hyperspectral imaging to establish a greenness prediction model before and after high-temperature stress, achieving early heat-stress monitoring and chlorophyll content prediction in ginseng. In practical monitoring, spectral reflectance varies across different growth stages; therefore, appropriate vegetation indices or models should be selected according to the specific developmental stage of the plant.

#### 3.3.2. Water Stress

Water stress can severely affect the growth of medicinal plants. Drought reduces biomass, triggers water loss, and causes osmotic imbalance within the plants [61,62], while waterlogging inhibits root respiration and leads to the accumulation of harmful substances [63]. Remote sensing technology can detect early stress by capturing the spectral responses of medicinal plants under different water conditions, providing technical support for timely field management. Drought-induced changes in leaf pigments can be observed in the wavelength range of 400–700 nm. Some researchers have explored the feasibility of using ultraviolet–near-infrared spectroscopy to monitor the drought effects on *Lavandula stoechas*, *Cistus laurifolius* and *Pistacia lentiscus* [64,65], laying the research foundation for stress monitoring of these plants. Cotton, an important economic crop, also has recognized medicinal applications, with its roots, shells, and seeds traditionally used for cough relief, asthma alleviation, and stomach warming. Under waterlogging conditions, Wu et al. [66] simulated varying degrees of waterlogging in cotton during the flowering stage and established a remote sensing inversion model based on the photochemical reflectance index (PRI), confirming the feasibility of hyperspectral technology in monitoring dynamic photosynthetic responses under excess water stress. Overall, remote sensing monitoring of water stress in medicinal plants usually requires a comprehensive assessment that combines plant growth status, photosynthetic indicators, and spectral characteristics.

#### 3.3.3. Heavy Metal Stress

Heavy metal stress inhibits medicinal plants’ growth, restricts root development, reduces yield, and exacerbates oxidative stress responses, leading to the accumulation of toxic compounds [67,68]. Medicinal plants are particularly sensitive to heavy metal contamination in soil and water, which directly affects both their growth and quality formation [69]. Remote sensing technologies enable the monitoring of heavy metal stress by exploiting correlations between metal concentrations and plant spectral characteristics. Chen et al. [70] analyzed the hyperspectral characteristics of *Celosia argentea* under manganese stress and found that with increasing manganese concentration, the chlorophyll absorption features in the red and blue regions became progressively shallower, while the green peak between them flattened. Moreover, the linear decrease in spectral slope provided an accurate indicator of stress intensity.

However, the underlying mechanisms linking physiological responses to spectral characteristics under heavy metal stress remain insufficiently understood, highlighting the need for further investigation into plant–stress–spectral interactions.

#### 3.3.4. Pest and Disease Stress

Pest and disease stress can significantly reduce both the yield and quality of medicinal plants, making timely monitoring essential for effective management [71]. Hyperspectral remote sensing technology can capture subtle differences in spectral reflectance and clearly identify beneficial changes in plant growth and development. Zheng et al. [72] extracted characteristic wavelengths and spectral indices from canopy spectra and used Fisher’s discriminant analysis to establish a detection model for the infection level of *Phyllostachys edulis*, achieving a discrimination accuracy of 84.4%. Zhao et al. [73] integrated UAV-based remote sensing with ground measurements to collect canopy spectral data of *Lycium chinense*, and constructed a fully connected neural network model that achieved prediction accuracies of 94.86% and 96.82% for *Bactericera gobica* and *Aceria pallida*, respectively. Zhao et al. [74] used visible spectral data and an SVM model to accurately identify early-stage infection in *Eleutherococcus senticosus*, demonstrating the potential for automated disease diagnosis. Current approaches primarily rely on hyperspectral data-driven models to classify diseased plants, assess disease severity, and enable early detection, thereby supporting more effective prevention and control strategies.

However, accurate identification of pest and disease stress in medicinal plants remains a major challenge, particularly when different stressors induce similar physiological responses. Although hyperspectral data provide continuous spectral information and greater sensitivity compared to other non-destructive techniques, their discriminative capability is fundamentally constrained by the complexity of plant stress responses. This limitation is reflected in the phenomena of “same spectrum from different stressors,” where different pests or diseases produce similar spectral features, and “different spectra from the same stressor,” where the same stressor induces variable spectral responses within the same plant. As a result, relying solely on spectral features is often insufficient for precise stress discrimination. Future research should therefore focus on integrating spectral data with physiological and pathological indicators, as well as incorporating multi-source observations, to enhance the reliability and specificity of pest and disease monitoring in medicinal plants. Representative studies on stress monitoring in medicinal plants are summarized in Table 4.

### 3.4. TCM Quality Assessment

#### 3.4.1. TCM Identification

Spectroscopy-based techniques, particularly HSI, have been widely applied to the identification of TCM, enabling the discrimination of authenticity, geographical origin, and growth age. Unlike large-scale remote sensing applications, these approaches are typically conducted at the sample level, where spectral responses primarily reflect variations in chemical composition and microstructural characteristics of TCM.

HSI provides contiguous spectral information, along with spatial context, allowing for the comprehensive characterization of samples. It has demonstrated strong capability in a range of identification tasks, including origin discrimination, authenticity verification, detection of fungal contamination, purity assessment, differentiation of medicinal parts, and estimation of growth years [42,75,76,77,78,79]. Representative methods and their corresponding performance are summarized in Table 5.

To achieve such performance, the analytical framework typically integrates spectral preprocessing, feature representation, and model construction, enabling classification via machine learning or deep learning approaches with generally high discriminative performance (often >90% accuracy).

Despite these advances, the discriminative capability of hyperspectral data is intrinsically governed by subtle variations in chemical composition, posing inherent challenges for distinguishing samples with highly similar constituents. Consequently, recent research has increasingly emphasized the integration of spectral and spatial features as an effective strategy to enhance model robustness and discrimination capability.

#### 3.4.2. TCM Component Inversion

Spectroscopy-based approaches enable the development of component inversion models by establishing relationships between chemical constituents and their corresponding spectral reflectance characteristics, thereby facilitating the quantitative retrieval of target components in TCM. HSI, in particular, has been widely adopted due to its ability to capture continuous spectral information associated with chemical composition.

Existing studies have demonstrated the effectiveness of both traditional machine learning and deep learning methods in component inversion, particularly for single-compound quantification. For instance, Liu et al. [80] developed a nonlinear CARS-SPA-LSSVM model based on hyperspectral data to estimate total phenolic content in honeysuckle. Representative methods and their performance are summarized in Table 6 [81,82,83,84].

Current research predominantly focuses on individual compounds, while the synergistic effects among multiple bioactive components remain insufficiently explored. From a spectral perspective, multi-component systems introduce additional complexity, as overlapping absorption features and nonlinear interactions among constituents obscure distinctive spectral signatures.

Recent efforts have begun to address this limitation by enabling simultaneous prediction of multiple components. For example, Wang et al. [85] integrated HSI with a temporal convolutional network-attention (TCNA) model to achieve rapid and simultaneous prediction of six rare ginsenosides in *Panax ginseng*, achieving strong performance (R^2^_P_ > 0.890 and RPD > 3.0). This approach demonstrates the feasibility of multi-component analysis using deep learning.

Furthermore, the generalizability of inversion models is constrained by the complex coupling between spectral responses and chemical composition. In multi-component systems, overlapping absorption features and nonlinear interactions lead to spectral ambiguity and reduced sensitivity, rendering the inversion inherently ill-posed. Addressing these issues requires deeper insight into spectral–chemical interaction mechanisms and the development of models capable of disentangling mixed signals and capturing inter-component dependencies.

## 4. Overview and Prospects

### 4.1. Overview

Over the past two decades, remote sensing research on medicinal plants has evolved from early species identification toward a comprehensive framework encompassing resource investigation, planting monitoring, stress monitoring, and quality assessment. The current state of research and its principal limitations can be summarized as follows.

Regarding data sources, a complementary sensing framework has taken shape, integrating satellite-, UAV-, and ground-based remote sensing. Satellite platforms have enabled macroscale mapping of medicinal plant distributions, UAV-based systems have permitted individual plant-level observation, and ground-based spectroscopy allows detailed measurement of specific medicinal organs. Nevertheless, most studies continue to rely on a limited range of data sources, and active sensing modalities such as SAR and LiDAR remain underutilized, constraining monitoring capacity in persistently cloudy regions and structurally complex habitats.

Hyperspectral remote sensing has made non-contact quality evaluation feasible, and models linking spectral reflectance to chlorophyll, moisture, and selected secondary metabolites have been established. However, model construction continues to rely predominantly on empirical statistical approaches, and ill-posed inversion problems arising from spectral ambiguity remain a persistent challenge. Quality assessment efforts tend to focus on individual compounds, with the mechanistic relationship between spectral response and multi-component pharmacological efficacy remaining incompletely understood.

Regarding application scope, remote sensing has been applied to national TCM resource surveys and geo-authentic production area assessment. However, continuous full life-cycle monitoring remains relatively scarce, disease monitoring is largely confined to post-event assessment, and the absence of standardized monitoring protocols continues to limit integration into precision agriculture frameworks.

### 4.2. Prospects

**Figure 4:**
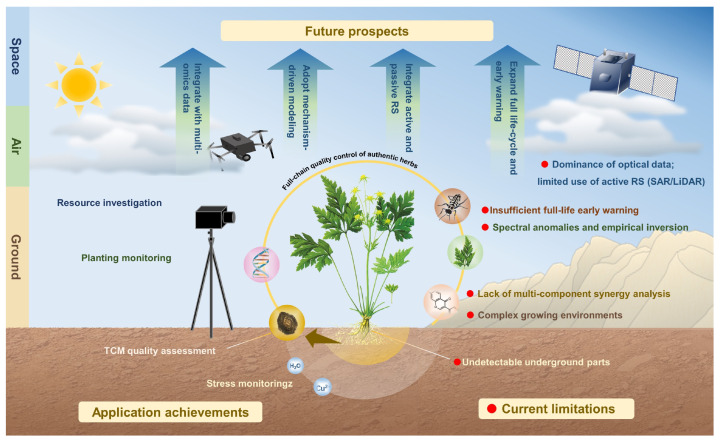
Application of remote sensing technology in medicinal plant monitoring and quality assessment.

Building on the evidence reviewed, several directions appear worthy of further investigation (Figure 4).

Strengthening the integration of active and passive remote sensing within multi-platform frameworks may help address the limitations of optical sensors in cloudy regions and complex habitats. The complementary advantages of SAR and LiDAR, when combined with optical data, could potentially improve resource characterization in heterogeneous environments.

Future quality inversion research may benefit from shifting toward mechanism-informed modeling. Investigating the spectral response mechanisms of secondary metabolites and incorporating physiological prior knowledge as model constraints could help alleviate ill-posed inversion problems. Exploring indirect spectral associations for constituents that are not directly detectable represents a potentially productive avenue.

Extending monitoring toward full life-cycle coverage and early warning applications represents a practically significant objective. Multi-scale inversion models may contribute to quality control and origin authentication as tools complementary to established analytical methods. Long-term satellite time-series data may inform habitat suitability modeling under projected climate scenarios. Coupling thermal infrared and radar observations for early disease detection shows preliminary promise.

Finally, integrating remote sensing-derived phenotypic data with multi-omics technologies may offer broader insights into the biosynthetic determinants of medicinal plant quality [86]. Advances in deep learning and foundation models may further improve data interpretation efficiency. Realizing this potential will require sustained investment in benchmark dataset construction, cross-environment model validation, and the development of interpretable frameworks connecting spectral observations to underlying biological processes.

## Figures and Tables

**Table 1:** Inclusion and exclusion criteria for study selection.

Subjects	Criteria Description
Inclusion criteria	The study involves medicinal plants as the primary research subject
	The study employs remote sensing technology (e.g., satellite imagery, UAV, hyperspectral or multispectral sensing) as a core method
	The study addresses at least one of the following topics: resource survey and distribution mapping, growth and cultivation monitoring, habitat and ecological environment assessment, or medicinal quality evaluation
	The study provides sufficient methodological description to allow assessment of the remote sensing platform, sensor type, or data analysis approach used
Exclusion criteria	The subject is not related to medicinal plants (e.g., non-medicinal crops, animals, or mineral substances)
	Remote sensing is not the primary technical approach (e.g., studies relying solely on laboratory chemical analysis, molecular biology, or field surveys without remote sensing data)
	Duplicate publications or studies with overlapping datasets, where only the most comprehensive version is retained
	The study does not fall within any of the four core application scenarios defined in this review

**Table 2:** Representative studies on the investigation of medicinal plant resources.

Application	Target Species	Data Source	Feature Extraction/Selection Methods	Model/Analysis Methods	Reported Performance (Best Result)	References
Resource survey and estimation	*Panax ginseng*	Satellite remote sensing (Landsat7 ETM, Spot, QuickBird)	N/A	Visual interpretation	ETM: OA = 90%; Fusion: OA = 97%; QuickBird: OA = 100%	[14]
Plot extraction and area estimation	*Polygonatum cyrtonema*	Satellite remote sensing (ZY-3)	Spectral bands	SVM	OA = 98.98%	[24]
Identification and classification	*Lamiophlomis rotata*	Airborne remote sensing (UAV)	RGB bands	Mask R-CNN (ResNet-101)	AP = 89.34%	[44]
Identification and classification	*Artemisia argyi*	Satellite remote sensing (GF-1, GF-6)	Spectral features, red-edge indices, time series	RF (GF-1 and GF-6 combined)	OA = 92.73%	[47]
Identification and classification	*Glycyrrhiza uralensis*	Satellite remote sensing (Sentinel-2)	Phenological indices (NDPVI, NDVWI)	Decision tree	OA = 83.85%	[48]
Plot extraction and area estimation	*Panax notoginseng*	Satellite remote sensing (GF-4)	Spectral features, vegetation indices, texture features	RF (pixel-based)	OA = 95.2%	[49]
Plant classification and extraction	*Callicarpa nudiflora*	Satellite remote sensing (WorldView-3)	Spectral features, vegetation indices, texture features; PCA	RF (optimized feature space)	OA = 90.4%	[50]

Notes: N/A: not applicable. Full names of the abbreviations in the tables are provided in the abbreviation list at the end of this paper.

**Table 3:** Representative studies on planting monitoring of medicinal plants: environment and growth/physiology.

Application	Target Species	Data Source	Feature Extraction/Selection Methods	Model/Analysis Methods	Reported Performance (Best Result)	References
Chlorophyll inversion	*Panax ginseng*	Ground-based remote sensing (Laboratory HSI system)	Vegetation indices; SPA	RF	RMSE = 1.1568, MAE = 0.9936 (Validation set)	[40]
Chlorophyll inversion	*Glycyrrhiza uralensis*	Airborne remote sensing (UAV), Ground-based remote sensing	CARS, GA, SPA	PLSR, XGBoost	Ground-based: GA-XGBoost, R_val_^2^ = 0.95 UAV-based: CARS-PLSR, R_val_^2^ = 0.83	[52]
Chlorophyll retrieval	*Syringa oblata*	Ground-based remote sensing (Specim IQ handheld HSI system)	RF	VR	R_cal_^2^ = 0.9442, R_val_^2^ = 0.9517	[53]
Chlorophyll distribution	*Dimocarpus longan*	Ground-based remote sensing (HyperSIS HSI system)	PCA (wavelengths), GLCM (textures), CNN (structures)	ICA-DNNs (multi-source data fusion)	R_cal_^2^ = 0.836, R_val_^2^ = 0.821	[54]
Nitrogen detection	*Dendrobium nobile*	Ground-based remote sensing (Gaiafield Pro-V10 HSI system)	CARS	Bagging	R_cal_^2^ = 0.908, R_val_^2^ = 0.935	[55]
Soil composition inversion	*Panax ginseng*	Laboratory HSI system (IMPERX 1920 × 1080 visible–near-infrared camera, Guohui 640 × 512 SWIR camera)	GA	ELM BPNN	SAS: ELM, R_val_^2^ = 0.88, RMSE = 28.19) (SM) BPNN: R_val_^2^ = 0.89, RMSE = 3.16	[56]

Notes: Full names of the abbreviations in the tables are provided in the abbreviation list at the end of this paper.

**Table 4:** Representative studies on stress monitoring relevant to medicinal plants.

Application	Target Species	Data Source	Feature Extraction/Selection Methods	Model/Analysis Methods	Reported Performance (Best Result)	References
Heat stress	*Panax ginseng*	Ground-based remote sensing (Fluorescence hyperspectral imaging system, 683–745 nm)	ANOVA	PLSR	R_cal_^2^ = 0.91 R_val_^2^ = 0.90	[41]
Drought stress	*Boehmeria nivea*	Airborne remote sensing (UAV)	Vegetation indices	PLSR	R_cal_^2^ = 0.885 R_val_^2^ = 0.778	[60]
Waterlogging	*Gossypium hirsutum*	Ground-based remote sensing (ASD FieldSpec 4 portable field spectroradiometer, 350–2500 nm)	Spectral parameter	Simple linear regression	*p* < 0.01 (All R^2^ values passed the significance test at the 0.01 level)	[66]
Heavy metal pollution	*Celosia argentea*	Ground-based remote sensing (ASD FieldSpec 4 portable field spectroradiometer, 400–1200 nm)	Green peak area, red-edge position shift, and specific band areas; PCA	Multiple linear stepwise regression	R^2^ > 0.84 (For most metal element models)	[70]
Plant disease	*Acanthopanax senticosus*	Laboratory HSI system (ImSpector N10E hyperspectral imager, 380–1000 nm)	PCA	SVM (Polynomial kernel)	OA = 92.77%	[72]
Plant disease	*Lycium chinense*	Airborne remote sensing (UAV)	Vegetation indices, sensitive bands	Fully connected neural network	OA = 96.82%	[73]

Notes: Full names of the abbreviations in the tables are provided in the abbreviation list at the end of this paper.

**Table 5:** Representative studies on TCM identification using hyperspectral sensing.

Application	Target Species	Data Source	Feature Extraction/Selection Methods	Model/Analysis Methods	Reported Performance (Best Result)	References
Foreign material detection	*Ziziphus jujuba* (Semen Ziziphi Spinosae)	Laboratory HSI system (SPECIM ImSpector-V10E; 400–1000 nm)	PCA, CARS, SPA	1D-CNN	R_cal_^2^ = 0.99 R_val_^2^ = 0.98	[42]
Origin identification	*Glycyrrhiza uralensis*; *G. inflata*; *G. glabra* (Glycyrrhizae Radix et Rhizoma)	Laboratory HSI system (SPECIM ImSpector-V10E; VNIR 435–898 nm; SWIR 898–1600 nm)	SPA	SVC	Accuracy: 96.44% (cal) 96.84% (val)	[75]
Authentication	*Ziziphus jujuba* (Semen Ziziphi Spinosae)	Laboratory HSI system (Hangzhou Caipu Technology Co., Ltd., FS-13; 400–1000 nm)	CARS	1D-CNN	Accuracy: 99.80% (cal) 99.50% (val)	[76]
Mildew degrees	*Lonicera japonica* (Honeysuckle)	Laboratory HSI system (Inno-Spec IST50–3810, 371–1024 nm)	UVE	ELM	Accuracy: 99.25% (cal) 96.97% (val)	[77]
Medicinal parts recognition	*Panax ginseng* (Red ginseng)	Laboratory HSI system (SPECIM ImSpector-V10E; 898–1751 nm)	N/A	PLS-DA;	Accuracy for roots: 96.79% Accuracy for main stem: 95.94%	[78]
Growth year identification	*Panax ginseng*	Laboratory HSI system (N/A; VNIR 400–1000 nm, SWIR 930–2500 nm)	N/A	FC-CNN	Accuracy: 71.20%/64.10%/67.70% (Three sets of accuracy based on different input bands)	[79]

Notes: N/A: not applicable. Full names of the abbreviations in the tables are provided in the abbreviation list at the end of this paper.

**Table 6:** Representative studies on component inversion of TCM using hyperspectral sensing.

Application	Target Species	Data Source	Feature Extraction/Selection Methods	Model/Analysis Methods	Reported Performance	References
Total phenolic content prediction	*Lonicera japonica* (Honeysuckle)	Laboratory HSI system (Inno-Spec IST50-3810; 371–1024 nm)	UVE, CARS, SPA	PLSR	R_cal_^2^ = 0.981 R_val_^2^ = 0.978	[80]
Multiple components’ content prediction	*Lycium barbarum* (Goji)	Laboratory HSI system (Norsk Elektro Optikk, HySpex VNIR 1800 and SWIR 384; 948.72–2512.97 nm)	CAM, SAM	1DCNN	CSAM-1DCNN: R^2^ > 0.93 (multi-task prediction)	[81]
Rosmarinic acid content prediction	*Ocimum basilicum*	Laboratory HSI system (Cubert GmbH Ultris 5; 400–850 nm)	AdaBoost, XGBoost, LightGBM	AdaBoost, XGBoost, LightGBM	XGBoost: R_cal_^2^ = 0.983 R_val_^2^ = 0.744	[82]
Moisture content prediction	*Ziziphus jujuba* (Semen Ziziphi Spinosae)	Laboratory HSI system (N/A; 380–1030 nm)	Subregional voting	PLSR; BPNN; CNN	CNN: R_val_^2^ = 0.99 PLSR: R_val_^2^ 0.98 BPNN: R_val_^2^ = 0.83	[83]
Total flavonoid and total polysaccharide content prediction	*Anoectochilus formosanus*	Laboratory HSI system (Vis/NIR HSI, Sichuan ShuangliHepu Technology Co., Ltd.; 400–1000 nm)	Selecting effective wavelength based on correlation	PLSR; SVM	PLSR: R_cal_^2^ = 0.9995 R_val_^2^ = 0.9978 SVM: R_cal_^2^ = 0.9996 R_val_^2^ = 0.9987	[84]

Notes: N/A: not applicable. Full names of the abbreviations in the tables are provided in the abbreviation list at the end of this paper.

## Data Availability

No new data were created for this article.

## Supplementary Materials

The following supporting information can be downloaded at https://www.mdpi.com/article/10.3390/s26082465/s1: Table S1: Summary of the relevant literature.

## Abbreviations

Adaboost	Adaptive Boosting
ANOVA	Analysis of Variance
Bagging	Bootstrap Aggregating
BPNN	Back Propagation Neural Network
CAM	Channel Attention Module
CARS	Competitive Adaptive Reweighted Sampling
CNKI	China National Knowledge Infrastructure
CNN	Convolutional Neural Network
ELM	Extreme Learning Machine
ETM	Enhanced Thematic Mapper
FC-CNN	Fully Connected–Convolutional Neural Network
GA	Genetic Algorithm
GF-1/4/6	GaoFen-1/4/6 Satellite (China)
GLCM	Gray Level Co-occurrence Matrix
HSI	Hyperspectral Imaging
ICA-DNNs	Independent Component Analysis–Deep Neural Networks
LiDAR	Light Detection And Ranging
LightGBM	Light Gradient Boosting Machine
LSSVM	Least Squares Support Vector Machine
MAE	Mean Absolute Error
Mask R-CNN	Mask Region-Based Convolutional Neural Network
NDPVI	Normalized Perennial Vegetation Index
NDVI	Normalized Difference Vegetation Index
NDVWI	Normalized Wilting Index
OBIA	Object-Based Image Analysis
PCA	Principal Component Analysis
PLS-DA	Partial Least Squares Discriminant Analysis
PLSR	Partial Least Squares Regression
R^2^	Determination Coefficient
R_cal_^2^	Determination Coefficient for Calibration Set
R_val_^2^	Determination Coefficient for Validation Set
OA	Overall Accuracy
AP	Average Precision
RF/RFR	Random Forest/Random Forest Regression
RMSE	Root Mean Square Error
RPD	Residual Predictive Deviation
RVI	Ratio Vegetation Index
SAM	Spectral Attention Module
SAR	Synthetic Aperture Radar
SAS	Soil-Available Silicon
SM	Soil Moisture
SPA	Successive Projections Algorithm
SVM	Support Vector Machine
SWIR	Short-Wave Infrared
TCM	Traditional Chinese Medicine
TCNA	Temporal Convolutional Network-Attention
UAV	Unmanned Aerial Vehicle
UVE	Uninformative Variable Elimination
VI	Vegetation Index
VNIR	Visible and Near-Infrared
VR	Voting Regression
XGBoost	Extreme Gradient Boosting
